# The combination of gas-phase fluorophore technology and automation to enable high-throughput analysis of plant respiration

**DOI:** 10.1186/s13007-017-0169-3

**Published:** 2017-03-21

**Authors:** Andrew P. Scafaro, A. Clarissa A. Negrini, Brendan O’Leary, F. Azzahra Ahmad Rashid, Lucy Hayes, Yuzhen Fan, You Zhang, Vincent Chochois, Murray R. Badger, A. Harvey Millar, Owen K. Atkin

**Affiliations:** 1 ARC Centre of Excellence in Plant Energy Biology, Research School of Biology, Building 134, The Australian National University, Canberra, ACT 2601 Australia; 2 Bayer CropScience SA-NV, Technologiepark 38, 9052 Gent (Zwijnaarde), Belgium; 3 ARC Centre of Excellence for Translational Photosynthesis, Building 134, The Australian National University, Canberra, ACT 2601 Australia; 4 Australian Research Council Centre of Excellence in Plant Energy Biology, University of Western Australia, 35 Stirling Highway, Crawley, WA 6009 Australia

**Keywords:** Dark respiration, Fluorophore, Gas-exchange, High-throughput, Oxygen consumption, Oxygen electrodes, Respiration, Respiratory flux, Respiratory quotient

## Abstract

**Background:**

Mitochondrial respiration in the dark (*R*_dark_) is a critical plant physiological process, and hence a reliable, efficient and high-throughput method of measuring variation in rates of *R*_dark_ is essential for agronomic and ecological studies. However, currently methods used to measure *R*_dark_ in plant tissues are typically low throughput. We assessed a high-throughput automated fluorophore system of detecting multiple O_2_ consumption rates. The fluorophore technique was compared with O_2_-electrodes, infrared gas analysers (IRGA), and membrane inlet mass spectrometry, to determine accuracy and speed of detecting respiratory fluxes.

**Results:**

The high-throughput fluorophore system provided stable measurements of *R*_dark_ in detached leaf and root tissues over many hours. High-throughput potential was evident in that the fluorophore system was 10 to 26-fold faster per sample measurement than other conventional methods. The versatility of the technique was evident in its enabling: (1) rapid screening of *R*_dark_ in 138 genotypes of wheat; and, (2) quantification of rarely-assessed whole-plant *R*_dark_ through dissection and simultaneous measurements of above- and below-ground organs.

**Discussion:**

Variation in absolute *R*_dark_ was observed between techniques, likely due to variation in sample conditions (i.e. liquid vs. gas-phase, open vs. closed systems), indicating that comparisons between studies using different measuring apparatus may not be feasible. However, the high-throughput protocol we present provided similar values of *R*_dark_ to the most commonly used IRGA instrument currently employed by plant scientists. Together with the greater than tenfold increase in sample processing speed, we conclude that the high-throughput protocol enables reliable, stable and reproducible measurements of *R*_dark_ on multiple samples simultaneously, irrespective of plant or tissue type.

**Electronic supplementary material:**

The online version of this article (doi:10.1186/s13007-017-0169-3) contains supplementary material, which is available to authorized users.

## Background

Mitochondrial respiration (*R*) is an essential physiological process in plants required for most energy-dependent metabolic processes. In mature leaves, *R* takes place in darkness (*R*_dark_) and in the light, and is central to processing of carbon assimilates and nitrogen assimilation [1], while also supporting the energy requirements of phloem loading and maintenance processes (e.g. protein turnover and membrane transport) [2–6]. Respiration is also central to the functioning of roots, providing the energy needed for biosynthesis, nutrient uptake and assimilation, as well as maintenance processes [7]. As such, genotypic and/or environmentally-induced variations in leaf and root *R* play a crucial role in determining growth/survival of individual plants, and productivity/functioning of terrestrial ecosystems [8–10]. Because of this, there is a growing need to describe and predict variability in rates of plant *R*, which in turn requires provision of large-scale data sets on leaf and root *R.* Recent studies reporting on expanded global data sets of leaf *R*_dark_ and its *T*-dependence [11–13]—compiled over several years using slow, low-throughput gas exchange protocols—are a step forward. However, our understanding of fine-scale temporal, spatial and developmental variation in plant *R* remains limited, both for natural and managed ecosystems. Addressing the need for new, large-scale datasets on plant *R* will require development of rapid, high-throughput methods capable of overcoming current bottlenecks in data provision.

One area where there is an urgent need for data on plant *R* is within the agriculture industry, where more energy-efficient crops are needed to improve global food security. For wheat (*Triticum aestivum*), only 10–15% of photosynthetic carbon gain contributes to yield [14], demonstrating the untapped potential for improving energy use efficiency. 30–80% of daily carbon gain by photosynthesis is subsequently respired [15–18], with respiratory costs increasing with increasing temperature [19]. Given that the efficiency of ATP synthesis per unit of CO_2_ or O_2_ equivalents respired varies (reflecting engagement of phosphorylating and non-phosphorylating pathways of mitochondrial electron transport [20, 21]), there is potential to improve crop yields via selecting for efficient genotypes with reduced rates of *R* [22, 23]. Indeed, there is growing evidence that physiological screening on a large scale assists crop breeders in identifying beneficial genetic material [24]. However, recombinant inbred line (RIL) populations, diversity panels and/or the structured genetic populations used in genome wide association studies (GWAS) typically include many hundreds of plant variants. Studying these for respiratory traits will require thousands of respiratory measurements to be routinely made on material at the same time of day and developmental stage.

Comprehensive *R* datasets are also needed to improve modelling of respiratory fluxes in terrestrial ecosystems [9, 25–27]. Using standard leaf gas exchange methods, recent surveys have greatly increased our understanding of biome-to-biome variation in leaf *R*_dark_ [11–13]; our understanding of how sustained changes in the environment affect respiratory rates is also improving [11, 28–31]. Yet, limitations in available data (e.g. documenting environmental, developmental and/or temporal variations) restrict our ability to fully describe the complexity of plant *R* that occurs in nature. Similarly, respiratory measurements have been conducted in only a small fraction of extant terrestrial plant species, limiting our ability to explore evolutionary changes in plant energy use efficiency. Addressing these challenges requires development of high-throughput methods for quantifying respiratory fluxes of plants growing in natural ecosystem across the globe.

Protocols using O_2_-electrodes and infrared gas-analysers have dominated the measuring of plant *R*_dark_ for several decades (refer to Hunt [32] for a comprehensive review of each techniques application, advantages and disadvantages). The O_2_-electrode technique was popularised in the form of Clark-type O_2_-electrodes, being first applied to measure human blood O_2_ levels [33]. O_2_-electrodes are often used for measurements of root respiration [34–36] and to assess the impact of exogenous substrates, uncouplers and inhibitors on leaf slices, intact roots and isolated mitochondria [37–39]. While a series of O_2_ electrodes can be set up in parallel to perform respiratory measurements, in most cases a single electrode is used and each measurement takes an estimated 25–50 min to complete (see Table 1 for a comparison of measurement times associated with this and other methods).

**Table 1:** Measurement times required per sample for each of the *R*_dark_ techniques assessed

Technique	Step	Description	*T* (min)
Fluorophore	Calibration	Purge tubes of air using N_2_ gas or sodium dithionite	0.02–0.05
	Sample preparation	Dissect tissue (e.g. scalpel, scissors or leaf punch) and place in measuring tube	0.5–1
	Measurements	In general, slopes taken from 1 to 2.5-h. 186 samples per run. Note: more than 186 samples can be simultaneously measured but cycle time between O_2_ recordings will increase to >6-min, reducing resolution	0.8
	Total		1.3–1.9
O_2_-electrode	Calibration	Prepare and assemble electrodes, including application of membrane and electrode solution. Aerate calibration solutions and obtain zero and saturated O_2_ values after stabilisation of current	4–9
	Sample preparation	Dissect tissue and place inside cuvette and adjust plunger being careful not to introduce air pockets	1–2
	Measurements	Slopes taken after stabilisation of signal and before depletion of O2, usually within 10–40 min but dependent on sample	20–40
	Total		25–51
IRGA	Calibration	Change consumables (e.g. soda lime, desiccant, CO_2_ canister) and zero IRGA chambers	1–2
	Sample preparation	Select and clip measuring chamber onto leaf	0.5–1
	Measurements	Allow steady-state gas-exchange to be reached	10–15
	Total		11.5–18
MIMS	Calibration	Apply membrane and test membrane stability. Purge tube and inject known volumes of O_2_ and CO_2_. Record background consumption	5–10
	Sample preparation	Dissect tissue and place inside cuvette and air-seal cuvette	1–3
	Measurements	Allow signal to stabilise (usually 5 min) and record slope between 5 and 20 min	20
	Total		26–33

*T* (min) represents the estimated time it takes to measure a single sample in minutes. For example, if 20 samples can be measured without recalibration and it takes 20-min to calibrate, then the calibration *T* is 1-min

Infrared gas-analysers (IRGA) are also commonly used to measure rates of plant *R* (as respiratory CO_2_ efflux), exploiting the infrared absorption properties of CO_2_. The major benefit of the IRGA systems is that they can be portable and operate as a gas-phase/open system. Such systems have been extensively used in recent times for quantifying plant *R*_dark_ [12, 40–42], including specialised chambers for whole-plant *R*_dark_ [16, 19, 43]. While a few research teams have developed multiplex systems for single IRGA measurement of four to 12 samples [e.g. 44], most IRGA measurements are made individually, each requiring 10–20 min per sample (Table 1). Consequently, existing IRGA methods are unlikely to provide the high throughput capacity needed to screen for genetic variations in energy use efficiency and/or improved modelling of ecosystem gas exchange.

Less employed spectroscopy technology for detecting respiratory O_2_ and/or CO_2_ exchange include tuneable diode laser (TDL) spectroscopy [45] and cavity ring-down (CRDS) spectroscopy [46]. Mass spectrometry can also be used, with one example of a mass spectrometry technique being membrane inlet mass spectrometry (MIMS), a gas phase method that is used to discriminate between O_2_ and CO_2_ isotopes, enabling deeper insight into the photosynthesis/respiratory process [44, 47]. Although MIMS is beneficial in that it can discern gas isotopes, neither it nor the above spectroscopic approaches are high-throughput (Table 1). Similarly, calorimetry measurements of metabolic heat rate and respiratory fluxes [48, 49] while providing an opportunity to explore relationships between respiration and growth—are also not high throughput.

Using O_2_-sensitive fluorophores in combination with fibre-optic fluorescent detection mechanisms for measuring the O_2_ evolution of photosynthesis of illuminated leaf disks was occurring by the late 1990s [50]. The technique works by exciting a fluorophore, in most cases a metal porphyrin, whose fluorescence is sensitive to O_2_ quenching. The measured decay rate of the fluorescent emission is thus proportional to the partial pressure of O_2_ present [51, 52]. This technology is becoming a more common technique for detecting respiratory O_2_ consumption of biological samples ranging from bacterial plankton to benthic meiofauna [53, 54]. The power of this technology is that many tissue types of varying abundance can be simultaneously and accurately measured. For example, fluorophore technology has enabled multiple simultaneous measurements of leaf, root and seed respiratory rates [55]. The authors highlight the high-throughput and small tissue size capabilities of the technique, not achievable using conventional Clark-type electrodes, infrared gas-analyser, spectroscopy or calorimetry methods. Yet, take-up of fluorophore technology to facilitate high-throughput measures of plant *R* remains limited, reflecting the need for more straightforward sample preparation than was possible using the liquid-phase approach of Sew et al. [55]. By contrast, using fluorophore technology in a gas-phase medium is likely to lead to faster processing times and avoid technical issues, such as floating tissues and air-pockets. To date, automated gas-phase measurements of O_2_ consumption using fluorophore techniques for plants have primarily focused on large-scale analysis of seed germination [56, 57], with automated, high-throughput assessments of non-seed plant *R* yet to be attempted using gas-phase fluorophore approaches.

To address the urgent need for high-throughput measurements of plant *R*_dark_, we have trialled an approach for measuring respiratory O_2_ uptake which re-purposes equipment designed for seed germination assays and combines the advantages of: (1) fluorophore technology that can accurately measure changes in O_2_ partial pressure in small measuring volumes that are easily calibrated; (2) closed, gas-phase measurements, which require minimal preparation time; and, (3) an automated sampling mechanism, relying on robotics to take measurements of multiple samples within a short period of time. As part of our study, we compare multiple O_2_ consumption detection methodologies to ascertain the reliability and compatibility of the different approaches. Further, to illustrate the potential of the high-throughput fluorophore technology to accelerate our understanding of plant *R*_dark_, we report on: (1) a screen of *R*_dark_ in 138 genotypes of wheat (using >550 plants) that was conducted over a few days; and, (2) rapid assessments of respiration in leaf, stem and root tissues that enable whole-plant respiratory fluxes to be estimated by simultaneous analysis of individually dissected plants.

## Methods

### Plant material

The species used in this study were a grass (wheat—*Triticum aestivum*), a herb (thale cress—*Arabidopsis thaliana*) and an evergreen broadleaved tree (red river gum—*Eucalyptus camaldulensis*), enabling the method to be tested on a range of plant functional types. Considering its agricultural significance, *T. aestivum* was selected as the primary species of interest, and all experiments, including the high throughput practical applications, were undertaken on *T. aestivum*, with a sub-set of other experiments conducted using other tissues. All experiments took place at the Research School of Biology at the ANU, Canberra, Australia plants grown in organic potting mix, enriched with Osmocote^®^ OSEX34 EXACT slow-release fertiliser, following manufacturer’s instructions (Scotts Australia, Bella Vista, NSW) with an N/P/K ratio of 16:3.9:10. Plants were watered daily to field capacity. For experiments where roots were analysed, wheat plants were grown hydroponically in a nutrient solution consisting of 1.4 mM NH_4_NO_3_, 0.6 mM NaH_2_PO_4_·2H_2_O, 0.5 mM K_2_SO_4_, 0.2 mM CaCl_2_·2H_2_O, 0.8 mM MgSO_4_·7H_2_O, 0.07 mM Fe-EDTA, 0.037 mM H_3_BO_3_, 0.009 mM MnCl_2_·4H_2_O, 0.00075 mM ZnCl_2_·7H_2_O, 0.0003 mM CuSO4·5H_2_O, 0.0001 mM (NH_4_)_6_Mo_7_O_24_·4H_2_O, 0.000138 mM NH_4_VO_3_, and 0.0012963 mM Na_2_SiO_3_. A pH ranging from 5 to 6 was maintained by adding concentrated sulphuric acid or sodium hydroxide, and monitoring of pH using a portable pH meter (Rowe Scientific Pty. Ltd., NSW, Australia). The hydroponic solution was aerated continuously using Infinity AP-950 aquatic air pumps (Kong’s Pty Ltd, Ingleburn, Australia). Plants were grown at temperatures of 25/20 °C for *T. aestivum* and *E. camaldulensis*, in temperature controlled greenhouses with natural photosynthetically active radiation (PAR) of between 400 and 1200 μmol m^−2^ s^−1^. *A. thaliana* was grown at 22/15 °C in temperature-controlled growth chambers (Thermoline, Wetherhill Park, Australia) with a PAR of 200 ± 30 μmol m^−2^ s^−1^ and a 12:12 h light/dark photoperiod. For leaf dissection samples, broad-leaved *A. thaliana* and *E. camaldulensis* leaf tissue was extracted using brass coring tools of known diameter and for *T. aestivum* a set distance of leaf blade was dissected with a scalpel. Where sectioned, root segments were dissected transversely from base to tip.

### High throughput fluorophore measurements

A Q2 O_2_-sensor (Astec Global, Maarssen, The Netherlands) designed and marketed for seed germination assays was used to obtain automated, high-throughput fluorophore measurements of dark respiration from plant material. A custom-built frame covered in black cloth was used to maintain darkness during sample measurements. Plant material were freshly dissected and placed in empty tubes (1, 2 or 4 ml in volume) and hermetically sealed with specialised caps (Astec Global). The top surface of caps contained a fluorescent metal organic dye, sensitive to O_2_ quenching. A blue-spectrum LED excitation pulse (approximately 480 nm) onto the surface of caps, followed by emission detection in the red spectrum (approximately 580 nm), enables the O_2_ dependent decay in fluorescence signal to be quantified. The fibre optic fluorescence detection unit is attached to a robotic arm which sequentially measures vials placed in racks of 48 tubes each (or 24, 4 ml tubes). The machine can accommodate 16 racks allowing 768 samples (1 or 2 ml tubes) to be measured in a single run. The frequency of measurements was in most cases set to 4 min, enough time to measure approximately 180 samples (a minimum measurement frequency of 1-min is required). The Q2 O_2_-sensor is calibrated before each set of measurements by measuring a designated tube containing ambient air (designated 100% O_2_), and a tube purged of all O_2_ using a sodium dithionite solution, or alternatively purging the tube of air using N_2_ gas (designated 0% O_2_). Output is given as an O_2_ percentage, relative to the calibration readings.

Based on the ideal gas law, raw output as the % O_2_ relative to the air calibration tube was converted to absolute values of dark respiration rates (*R*_dark_) in moles of O_2_ s^−1^ using Eq. 1.

1 $$R_{dark} = \frac{{P_{o} VS}}{RT}$$

*P*_*o*_ equals 20.95, the partial pressure of ambient O_2_ in kPa (i.e. 20.95% of atmospheric pressure), and *V* equals the volume of the sample tube (1, 2 or 4 ± 0.2 ml tubes were used in this study). *S* refers to the slope of sample tubes O_2_ consumption, (as a % of air and subtracting the air calibration tube slope), from 1 to 2.5 h after the beginning of sample measurements, expressed as the % of O_2_ per second. *R* is the gas constant (8314 cm^3^ kPa K^−1^ mol^−1^) and *T* is the temperature in Kelvin (K). The final calculation of O_2_ consumption rates in moles s^−1^ were expressed on a leaf area (cm^2^) basis, calculated from the diameter of the leaf corer (for leaf disks) or ruler measurements for grass leaf sections. Alternatively, for whole-plant developmental partitioning measurements respiration was expressed on a fresh mass basis. To test technical reproducibility of the instrument, a chemical oxidation assay consisting of 100 mM of cysteine in 600 μL of buffered solution (50 mM Hepes, 10 mM MES pH 6.5, 200 μM CaCl_2_) was used, and the stabilised O_2_ consumption rate over a 2-h run was measured repeatedly. To test fluorophore sensitivity to O_2_ depletion, known volumes of pure CO_2_ gas were injected into tubes through a pin-hole created on the side of tubes and sealed with blu-tack (Bostik, Paris, France) immediately after the gas-injection. All measurements were made at a room temperature of 21.5 ± 1.0 °C.

### O_2_-electrode measurements

Respiratory consumption of O_2_ by leaves (3–42 mg fresh mass) or roots (56–214 mg fresh mass) were measured in the liquid-phase using Oxytherm Clark-type O_2_-electrode (Hansatech Instruments, Pentney, UK) in a 2 ml measuring volume. Electrodes were calibrated by bubbling water with compressed air for approximately 2-h to reach saturation followed by adding sodium dithionite to record O_2_ depleted signals. Leaf and root respiration was measured in a solution containing 20 mM Hepes (pH 7.2), 10 mM MES and 2 mM CaCl_2_, at 21.5 ± 1 °C. All measurements were made by dark adapting tissue for >30 min, submerging tissue in the Clark-type electrode cuvettes below measuring solution, with no obvious air pockets and continually stirring, and recording O_2_ consumption using Oxygraph Plus v1.02 software (Hansatech Instruments). The linear part of O_2_ consumption (approximately 10–30 min into each run) was used to calculate respiration rates.

### IRGA measurements

Infrared gas-analysis of CO_2_ efflux by respiring leaves was measured using a Licor 6400XT with a 3 × 2 cm chamber head ((LI-COR, Lincoln, Nebraska, USA) on >30 min dark-adapted leaves. Attached whole leaves were placed across the measuring chamber and chamber gaskets and measurements recorded after CO_2_ readings stabilised (~10–15 min). The flow rate was set to 300 µmol s^−1^, the block temperature set to ambient air temperature of 22 °C and the CO_2_ reference sample was set to 400 µmol mol^−1^, to match ambient air. The light source was turned off.

### Membrane inlet mass spectrometry

Dark-adapted wheat leaf disks (3 × 0.5 cm^2^ or 6 × 0.5 cm^2^) were placed in a 1 mL O-ring sealed cuvette containing only air and a polyethylene membrane sealed outlet attached to a mass spectrometer (MM6: VG, Winsford, UK). O_2_ (m/z = 32) and CO_2_ (m/z = 44) detection over a 20-min period was recorded. Prior to leaf disk samples being placed in the cuvette, N_2_ gas purging of the cuvette and injections of known volumes of O_2_ and CO_2_ allowed for conversion of mass detection signal to a gas concentration and the background consumption rate of O_2_ and CO_2_ by the mass spectrometer to be accounted for when determining leaf derived O_2_ consumption and CO_2_ evolution rates.

### Replication and statistical analysis

For all experiments four to six biological replicates, with a biological replicate considered as plant material from individual plants grown in separate pots, or containers (when grown hydroponically) were measured. For the comparison of respiratory techniques, two or more samples from each biological replicate were analysed by each technique and sampling was standardised by selecting a 2 cm long mid-section of young, healthy, fully expanded leaves, or in relation to root samples, a longitudinal section from base to tip of the longest root segment. A one-way ANOVA was used to determine significance between leaf O_2_ consumption techniques and Two-Sample *t*-tests for differences between leaf CO_2_ evolution techniques and root O_2_ uptake techniques.

## Results

### Technical and biological reliability and accuracy

**Fig. 1:**
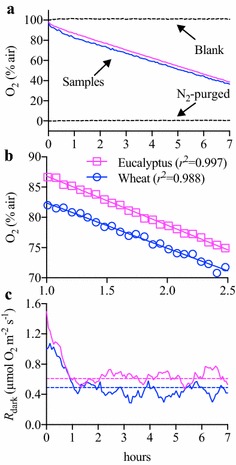
O_2_ consumption rates measured using the fluorophore technique. **a** O_2_ is given as a percentage of O_2_ in ambient air. A tube containing no sample (labelled as Blank) provided the baseline for no O_2_ consumption, while a tube devoid of all air (labelled as N_2_-purged) provided the baseline for total O_2_ consumption, and samples (*magenta* for Eucalyptus; *blue line* for wheat) depleted O_2_ within this range. **b** A higher resolution plot of individual data points over a 90-min period, from 1 to 2.5-h and linear regression analysis. **c** Respiration rates calculated from linear regression of O_2_ consumption using Eq. 1. Presented are *R*_dark_ calculated from a 1-h moving slope (solid lines), and *R*_dark_ calculated from the 1–2.5 h slope as shown in Panel b (*dashed horizontal lines*). Values are the means of four biological replicates for each species with the % of O_2_ measured every 4 min

The stability of the fluorescent oxygen concentration measurements performed using the Q2 is evident because control tubes containing either ambient air or no O_2_, gave long-term stable readings at 100 ± 5 or 0 ± 5%, respectively (Fig. 1a). The stability of O_2_ in the purged tubes demonstrates that the sample tubes were hermetically sealed, providing a closed system, necessary for accurately measuring O_2_ uptake. Nevertheless, we suggest periodically testing the accuracy of the calibration tubes, in case of drift over time, by placing 100% air and 0% O_2_ tubes amongst samples during a run. Measuring the spontaneous chemical oxidation of a cysteine solution in replicate vials assessed the technical reproducibility of the O_2_ consumption measurements. Analysis of 30 tubes in three separate experiments gave an average coefficient of variation of 8.1% (Additional file 1: Table S1).

When cut leaf material was placed inside the sample tubes, the fluorophore system was able to measure a consistent decline in O_2_ over a greater than 7-h period following an initial 1 h period of stabilization (Fig. 1a). The decline was linear in all species and tissues tested. The 90-min O_2_ consumption slope between 1 and 2.5-h had a mean *r* ^2^ of 0.99 across both species (Fig. 1b). Typically, the initial 0–30 min period of each run was associated with sharp declines in the O_2_ consumption slope. *R*_dark_ calculated from a 1-h moving average of slopes over 7-h was similar to the slope of O_2_ consumption over a set 90 min period between 1 and 2.5-h (presented as dashed horizontal lines in Fig. 1c). The O_2_ consumption slope between 1 and 2.5-h can therefore be used as a standard period for calculating *R*_dark_ across experiments.

**Fig. 2:**
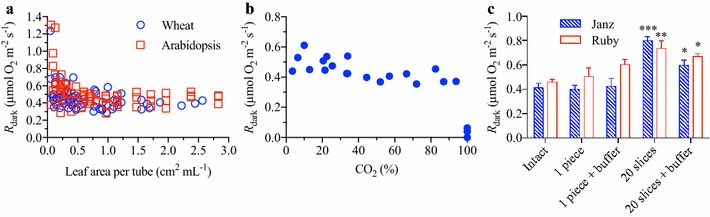
The influence of leaf tissue amount, CO_2_ concentrations, and mechanical wounding on the dark respiration rate (*R*_dark_) of wheat and Arabidopsis leaf tissue. **a** Differing amounts of leaf area in measuring tube volumes (cm^2^ mL^−1^) plotted against corresponding *R*_dark_ for wheat (*open blue circles*) and Arabidopsis (*open red squares*). **b** *R*_dark_ of wheat leaves sealed in measuring tubes with varying CO_2_ as a % of air. **c** The influence of mechanical wounding during sampling of leaf sections on the dark respiration rate (*R*_dark_) of two wheat cultivars, Janz and Ruby. The *R*_dark_ of a 2 × 0.5 cm transverse section of leaf and a same sized leaf sliced a further 20 times was compared to an intact leaf, which was not mechanically damaged. Alternatively, leaves that were cut were washed with a wounding buffer prior to *R*_dark_ measurements. The values are mean ± SE of four biological replicates. *significance at P < 0.05, ** at P < 0.01 and *** at P < 0.001, for a one-way ANOVA and a Dunnett’s multiple comparison test with the intact leaf set as the control

Respiratory rates per unit leaf area were independent of the amount of leaf material placed within a given tube volume, apart from exceedingly small tissue abundance of below 0.1 cm^2^ mL^−1^ (Fig. 2a). To test whether the signal was independent of CO_2_ concentration and linearly related to O_2_ concentration between 0 and 100% of atmospheric O2 known volumes of pure CO_2_ gas were injected and sealed in measuring tubes. The measured percentage of O_2_ in the tube declined linearly in close proximity to the expected values for the amount of air displaced by CO_2_ (Additional file 1: Fig. S1), validating that for the fluorophore in question, the O_2_ dependent fluorescence quenching is linear and independent of CO_2_ concentration. An increase in CO_2_ concentration was not inhibitory to *R*_dark_, evident in maintained *R*_dark_ when O_2_ was depleted to less than 40% of ambient levels, equivalent to the gas volume being >8% CO_2_, assuming a respiratory quotient of one. We provided further support of a lack of CO_2_ inhibition of *R*_dark_ by purging tubes containing wheat leaf samples with various concentrations of pure CO_2_ gas (Fig. 2b). Interestingly, replacing the volume of gas surrounding leaf material with as much as 90% CO_2_ did not lead to a substantial decline in *R*_dark_. When 100% of the air within a tube was replaced with CO_2_, *R*_dark_ did essentially stop, understandable considering no O_2_ would be available for respiration.

Although increased CO_2_ concentration was not inhibitory to *R*_dark_, heavy mechanical wounding of tissue resulted in higher *R*_dark_ (Fig. 2c). Intact wheat leaves versus a 2 × 0.5 cm transverse section from the middle of leaves (a ratio of 1:1, wounded boundary length to leaf area) did not exhibit significant differences in *R*_dark_ on an area basis (Fig. 2c). However, if the transverse section was further sliced into 20 smaller pieces (a 20-fold increase in the cut surface length to leaf area ratio), *R*_dark_ increased by as much as two-fold (Fig. 2c). Applying a buffered saline solution to the heavily wounded leaf partly mitigated the enhancement of *R*_dark_ by wounding. Thus it is important to reduce the amount of tissue exposed to mechanical damage when processing samples, to avoid the risk of artificially enhancing respiration rates.

### Comparisons between leaf gas-exchange methods

Considering the many methods currently in use for determining plant respiratory gas-exchange, and the need to ensure that the fluorophore system was giving comparable rates, we compared *R*_dark_ values generated using the fluorophore technology, the more conventional Clark-type O_2_-electrodes, Licor 6400 IRGA gas-exchange system, and membrane inlet mass spectrometry (MIMS). All of these techniques have varying degrees of difference in sample preparation and technical methodology that may influence the final respiratory rate recorded. For example, while we measured O_2_ consumption in the gas-phase using the fluorophore technique, O_2_-electrode measurements were made in aqueous-phase. Despite the IRGA measurements being made in gas-phase, measurements were of CO_2_ rather than O_2_ flux, and in an open gas-exchange system rather than the closed fluorophore system. Furthermore, IRGA measurements are made on intact not detached leaves. MIMS would be closest in methodology to the fluorophore technique in that both were measuring in the gas phase, in an essentially closed system. However, the MIMS system is not a completely closed system as the gradual leak of gasses through the semi-permeable membrane to the mass spectrometer would lead to changes in partial pressure and water vapour at the site of the leaf.

**Fig. 3:**
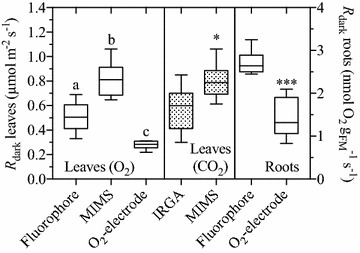
Comparisons of wheat dark respiration (*R*_dark_) measurements made using different experimental techniques. Leaf and root *R*_dark_ was calculated from O_2_ consumption (*open boxes*) and CO_2_ evolution rates (*hatched boxes*), measured using fluorophore quenching, membrane inlet mass spectrometry (MIMS), Clark-type O_2_-electrodes, and infrared gas analysis (IRGA). Whiskers of *box-plots* represent the 5–95 percentile. For leaf O_2_ analysis, letters indicate significant differences between techniques at *P* < 0.05, derived from a one-way ANOVA with a Tukey’s multiple comparison test. For leaf CO_2_ analysis and roots an unpaired t-test was performed and * indicates significance at *P* < 0.01, while ***significance at *P* < 0.001. All measurements were made at 21 ± 0.5 °C. The values are based on six biological replicates and greater than 12 technical replicates

Understandably, due to the aforementioned differences in methodology, calculations of *R*_dark_ using matching leaf or root material were significantly different between methods (Fig. 3). On an O_2_ basis, the conventional O_2_-electrode technique gave lower values, MIMS gave higher values, and the fluorophore values were intermediate. On a CO_2_ basis, MIMS measurements were significantly higher than IRGA measurements. MIMS, the only technique that can measure both O_2_ and CO_2_ concentrations, gave almost matching *R*_dark_ measurements on an O_2_ and CO_2_ basis, indicating a respiratory quotient near unity for darkened wheat leaf tissue. Root *R*_dark_ measurements in the gas-phase on the fluorophore system were significantly higher than in the liquid phase measured with O_2_-electrodes. Thus, while the fluorophore and IRGA approaches provide similar estimates of leaf *R*_dark_, both methods yield relatively lower estimated respiratory fluxes compared to MIMS; by contrast, the fluorophore approach yields relatively high values compared to liquid-phase Clark-type O_2_ electrode measurements.

### High-throughput analysis of respiration

Two studies were undertaken to verify the capabilities and versatility of automated O_2_ fluorophore technology for measuring high-throughput plant respiration in leaves and other plant tissues.

**Fig. 4:**
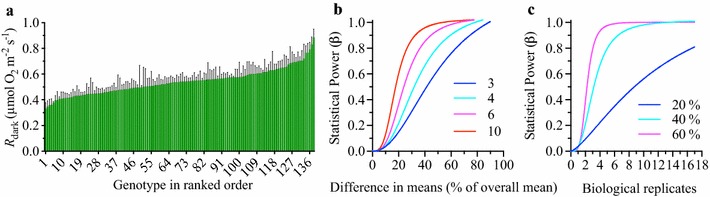
A high-throughput genotype and statistical power analysis of wheat dark respiration (*R*_dark_) of the youngest fully expanded leaf. **a** Leaf respiration in the dark (*R*_dark_) across 138 wheat cultivars grown under common conditions in a controlled environment growth room and measured at 21.5 ± 1 °C. The experiment was replicated on four occasions and the means (*green columns*) ± SE (*black bars*) of four biological replicates are presented. **b** Statistical power analysis of the wheat dataset with lines representing different numbers of biological replicates (3, 4, 6, or 10) plotted as a function of statistical power versus difference in genotype mean values. **c** Statistical power analysis of the wheat dataset with *lines* representing differences in mean values between genotypes equal to 20, 40 or 60% of the overall mean (equal to effect sizes of 1, 2 and 3 times the standard deviation) plotted as a function of statistical power versus biological replicates

For the first study we undertook a fully replicated experiment of leaf respiration in 138 wheat cultivars (Fig. 4). There were clear differences in *R*_dark_ among many genotypes, with a two-fold variation between the lowest and highest respiring cultivars (Fig. 4a). The wheat dataset was used to calculate the average standard deviation among biological replicates. As a proportion, the standard deviation was close to 20% of the overall mean *R*_dark_. This coefficient of variation was used to estimate the statistical power for future *t* test comparisons of *R*_dark_ between wheat lines as a function of replicate number and difference in means, using a false discovery rate of 5% (α = 0.05; not including corrections for multiple testing). Given the four biological replicates per genotype used in this 138-genotype study, there is sufficient statistical power [(1 − β) > 0.8] to consistently detect only large differences in *R*_dark_ between two lines equal to 50% of the mean (Fig. 4b). In a further example, to detect a 20% difference in mean respiration rates between any two wheat lines with the conventional statistical power target of (1 − β) = 0.8, 17 replicates would be appropriate (Fig. 4c). Of course, significant differences can still be detected with less replicates and less power, but given the high-throughput capacity of the fluorophore technique, appraisal of statistical power and appropriate biological replication can now be achieved, where previously, such high levels of replication were a barrier to experiments.

With the potential to run a single sample using the fluorophore system in less than 2 min (Table 1), a single replicate of all 138 genotypes could be processed in less than 4 h, and potentially, a fully replicated 138 genotype study could be achieved in a single day. The number of samples per day is limited by the capacity of the robotic system, and by the time taken to prepare samples. By comparison, the other techniques have significantly longer calibration, sampling and measurement times required to acquire a single measurement (Table 1). Hence, what can be undertaken in 8-h using the high-throughput fluorophore technique, would require a minimum of 83 equivalent hours, or as much as 200-h for other commonly used procedures to measure *R*.

**Fig. 5:**
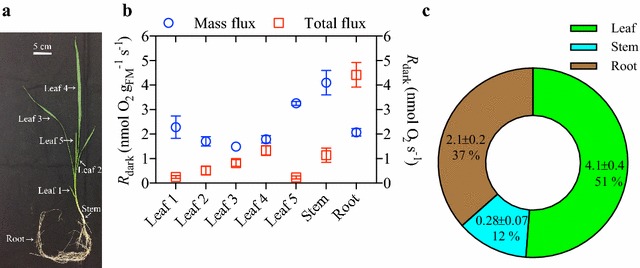
Wheat plants were harvested and separated into individual leaves, stem and roots, enabling an analysis of whole-plant dark respiration (*R*_dark_). **a** A representative image of a tiller with the roots, stem and leaf positions labelled. Leaf position 1 refers to the oldest leaf and position 5 refers to the newest emerging leaf. **b** Individual leaf, stem and root *R*_dark_ on a mass basis (Mass flux; plotted on the left-axis) and total flux basis (Total flux; plotted on the right-axis). **c** A pie diagram illustrating the partitioning of the total flux of *R*_dark_ between leaves as a whole, the stem and roots. The total plant leaf, stem and root fresh masses (g) are provided, as well as the % flux of total *R*_dark_. Values are based on the mean ± SE of six biological and pot replicates

The second study looked at whole-plant developmental partitioning of *R*_dark_ between leaves, stems and roots of 46-day-old wheat plants, which had reached the tillering stage of development (Additional file 1: Fig. S2). This type of experiment enables the quantitative attribution of total plant *R*_dark_ to different parts of the plant at a specific stage of development. The simultaneous measurement of a whole dissected plant saves on the need to combine rates over time from measurements made on different plants. Plants were dissected and the individual leaves (including both leaf blade and sheath), the stem and roots were separated. The *R*_dark_ of all separated tissues was measured for six entire plants simultaneously. Relative to healthy fully expanded leaves of a tiller; *R*_dark_ was slightly higher in the oldest and much higher in the youngest leaves, on a fresh mass (i.e. nmol O_2_ g^−1^ s^−1^) basis (Fig. 5a, b). Leaves of intermediate ages exhibited similar rates of mass-based *R*_dark_. The total *R*_dark_ for an entire leaf increased with age, presumably due to the increase in leaf size with stem and tiller developmental maturity. However, the total flux of O_2_ for the youngest leaf of the main stem or tiller was low (Fig. 5b), due to the smaller leaf size (Fig. 5a). When considered together, the total respiratory output of wheat foliage is dominated by healthy, relatively young, fully-expanded leaves (Fig. 5b) despite the oldest and youngest leaf of a stem or tiller having greater rates of *R*_dark_ on a mass basis. When considering the partitioning of *R*_dark_ between all tissues of the entire plant, leaves accounted for 51% (Fig. 5c), roots 37% and stems 12% of the total respiratory flux. Although the stem accounted for 12% of total *R* flux, it was only 4% of the entire fresh mass of the plant; however, stems had the highest mass based fluxes, likely due to energy expensive processes of cell division and elongation at the site of the apical meristem.

## Discussion

We demonstrate that using robotic fluorophore-based gas-phase measurements of O_2_ consumption in sealed tubes provides a simple yet reliable and reproducible means of measuring *R*_dark_ for a diverse range of plant tissue types and species. The technique differentiates itself from other conventional methods in that it significantly reduces the time required for sample preparation and has substantial simultaneous measuring capabilities, making the technique a truly high-throughput means for measuring respiration. We demonstrate the potential capabilities of the method by measuring *R*_dark_ of 138 wheat genotypes, and by measuring *R*_dark_ of all tissues of six mid-vegetative stage plants simultaneously. A comparison of *R*_dark_ in absolute terms, generated by different methodologies suggests variation in respiratory rates depending on technique employed, which should be considered when making direct comparisons between methods.

### Strengths and weaknesses of high-throughput fluorophore methods

There was an initial spike and rapid decline in respiratory activity within the first 30-min of measurements (Fig. 1b). We dark-adapted leaves for a minimum or 30-min prior to fluorophore analysis, so although it is common to find a spike in respiration of leaves following exposure to light within the initial 30-min post-illumination period [58], post-illumination bursts in respiration do not explain the findings. Furthermore, while the O_2_-electrode and MIMS measurements continuously recorded in a similar manner to the fluorophore system, neither approach showed the initial spike, followed by rapid decline in *R*_dark_ that was exhibited by the fluorophore approach (Fig. S3). Consequently, the first 60-min of each run were not used to calculate rates of *R*_dark_ in the genotypic and developmental studies; the initial stabilisation period, however, can be used as a dark-adaptation period if tissue is not dark-adapted prior to fluorophore experimentation.

CO_2_ has previously been postulated to inhibit cytochrome *c* oxidase (COX) activity [59]. Reports initially suggested that a doubling of current atmospheric CO_2_ (i.e. from 0.04% of atmospheric gas to 0.08%) reduced *R*_dark_ by 15–30% [60–62]. However, it was later discovered that CO_2_ inhibition of *R*_dark_ was mostly likely an artefact of the measuring techniques used to quantify respiratory CO_2_ release [63–65]. Our results show that CO_2_ accumulation does not inhibit *R*_dark_. In fact, even with CO_2_ concentrations surrounding the sampled tissue reaching more than 90% of the gas volume (a 450-fold increase in concentration relative to previously reported measurements), no substantial inhibition in respiration occurred (Fig. 2b). We therefore conclude that leaf *R*_dark_ is highly insensitive to CO_2_ accumulation over a course of several hours.

One factor that does seem to influence *R*_dark_ is mechanical wounding (Fig. 2c). Leaf wounding was thought to affect leaf respiration as far back as 1950 [66]. Increased *R*_dark_ with mechanical wounding is attributed to stimulation of the ATP/ADP ratio and activation of pyruvate kinase due to ion changes associated with wounding [67]. Pre-treatment by washing leaf samples with a buffered saline solution, the same as the measuring solution in liquid phase measurements, reduces any wounding effects on leaf *R* [38, 68]. We observed an increase in *R*_dark_ when a large proportion of the sample had a wounded edge, and a reduction in *R*_dark_ by applying wounding buffer, although not enough of a reduction to eliminate the wounded effect (Fig. 2c). However, minimal wounding did not significantly change *R*_dark_. Considering the time required to wash the sample tissue with a wounding solution, we suggest minimising as much as possible the mechanical wounding of tissue, rather than applying a wounding solution, if high-throughput sampling is desired. However, minimising mechanical wounding may require using larger volume tubes (e.g. moving from 1 to 4 mL tubes) to adequately fit sample tissue. By running a preliminary experiment, one could initially check for wounding effects and use the appropriate tissue size thereon after.

The limited effect of leaf wounding and lack of any inhibition to *R*_dark_ from CO_2_ accumulation resulted in respiration measurements being stable over a period of many hours (Fig. 1). The stability of *R*_dark_ for small leaf sections means that although the fluorophore technique we present is a closed-system that destroys the sampled tissue, a small sample of leaf collected in the field can be transported to the lab (making sure to keep detached leaves from desiccating), accurately representing in situ *R*_dark_. Thus, the fluorophore method can be considered as a pseudo non-destructive technique for high-throughput analysis for field experiments, as demonstrated below in the 138 wheat genotypes study we present.

### Comparisons between respiratory methods

Although Hunt [32] comprehensively compared the strengths and weaknesses of multiple photosynthesis and respiration measurement techniques, no study to our knowledge has directly compared the absolute values of *R* obtained from the same biological material but measured across multiple techniques. Determining if the fluorophore technique presented in our study is comparable with previously well-established methods is important. Firstly, if results are to be examined among studies that utilised different techniques, it must be established if the analysis is viable, or whether differences among studies are an artefact of measuring technique. Secondly, although in many cases only the relative differences in *R* between samples may be of interest (for example, the genotypic study we present here), in many circumstances, absolute *R* will be desired, such as for determining absolute photosynthesis, or modelling the impact of *R* on terrestrial carbon budgets. Hence, we directly compared fluorophore, O_2_-electrode, IRGA, and MIMS output (Fig. 3). We found differences did exist between the techniques, suggesting that comparing results between studies utilising different *R* measuring apparatus may not be appropriate, or at least with the caveat that comparisons may require cross-calibration of method. Differences in measurements based on either O_2_ consumption or CO_2_ evolution may be expected considering the respiratory quotient (*RQ*) will not necessarily be equal to 1 (i.e. respiratory CO_2_ release being equal to O_2_ uptake) if pure carbohydrates were not the only source of respiratory substrate, or the oxidation state of respiratory products differed, although a *RQ* of 1 is usually assumed for higher plants under non-stressed conditions [69]. Indeed, the simultaneous measurement of *R*_dark_ derived from O_2_ and CO_2_ exchange by MIMS gave close to matching values, supporting a *RQ* of 1, in contrast to a study of wheat leaves measured in the dark, 6-h into the light period (similar conditions to this study), which gave a *RQ* value of 1.8 ± 0.21 [70]. However, the study by Azcón-Bieto, Lambers and Day [70] used values of *R* determined separately using O_2_-electrode and IRGA systems, and since we found lower O_2_ based O_2_-electrode values relative to CO_2_ IRGA values, we emphasise that caution must be taken when comparing *R* calculated from different methodologies. Of note, the widely used IRGA gas-exchange system on intact leaves gave similar rates to the fluorophore results, suggesting the two techniques may be complementary. We did not undertake subsequent experiments to determine the specific reasons for variations in *R*_dark_ between the techniques compared, and it will be of interest to further explore the reasons for why the techniques vary in future studies.

### Genotypic and whole-plant analysis

Both a comprehensive genotype comparison and whole-plant respiratory balances were successfully obtained by use of the gas-phase automated fluorophore technique. Interestingly, a more than two-fold variation in *R* was observed between the 138 wheat genotypes (Fig. 4a). This demonstrates the inherent intra-specific divergence of *R* in *Triticum aestivum*, and a potential target for future yield improvements, if *R* not contributing to growth or yield can be minimised. Inherent differences in *R*_dark_ between species populations have previously been noted, such as in the ryegrass species *Lolium perenne*, attributed to adenylate limitations on glycolysis and varying ATP turnover rates between populations [71]. *R* was also highly variable among genotypes. This may not be considered surprising as leaf functional traits vary considerably among populations/genotypes within a given species. For example, a study of 13 common alpine species found that 30% of observable variance in measured traits, such as specific leaf area and leaf nitrogen content, was among populations/genotypes of a given species [72]. Similar results were found for species growing in a dry tropical forest [73]. Considering *R* is highly variable among genotypes within species, to gain sufficient statistical power a high level of replication is required (Fig. 4b, c), further supporting the benefit of the high-throughput fluorophore technique we present.

Our whole-plant respiratory analysis demonstrated the important effects of plant development on leaf *R* and partitioning of *R* between tissue types, as previously demonstrated in Arabidopsis by Sew et al. [55], which could be detrimentally ignored if the power of high-throughput respiratory analysis was not readily available. The results highlight the fact that, when measuring leaf, stem and root O_2_ uptake in the gas phase, leaf *R*_dark_ accounted for 51% of the entire *R* budget. In other words, close to half of all vegetative-stage wheat *R* occurs in non-leaf tissue, a finding reported for previous studies that quantified whole-plant CO_2_ fluxes [15–19]. Yet, we tentatively suggest that the majority of plant *R* reports would focus entirely, or predominantly on leaf *R*. Furthermore, the oldest and newest emerging leaves had considerably higher mass-based rates of *R*_dark_ than intermediate aged leaves. In regards to the latter, this is presumably due to the added cost of growth *R* as well as maintenance *R* for newly emerging leaves [6]. The spike in *R* for the oldest leaves may reflect the costs associated with senescence, such as an energy expensive remobilisation of nutrients from the senescing leaf to other parts of the plant. For example, in oats (*Avena sativa*), promotion of senescence of leaves by withholding light leads to a greater than two-fold increase in O_2_ consumption, attributed to decoupling of *R*_dark_ from oxidative phosphorylation, and amino-acid and soluble sugar liberation during senescence [74].

## Conclusions

The high-throughput and tissue size versatility of the experiments we conducted highlight the comparative advantages of an automated gas-phase system, over other systems based on the same technology but reliant on aqueous-phase and limited sample tubes and volumes. Although aqueous-phase fluorophore systems may be relatively high-throughput when compared to the older technology of Clark-type O_2_ electrodes, liquid-phase measurements still require extensive time in preparation of solutions, dispensing of solutions, and delicate sample positioning or sufficient stirring to facilitate O_2_ movement to the sensor [e.g. 75]. We processed 138 samples, from tissue harvesting to initial O_2_ uptake measurements, in a period of less than 2-h, which was possible due to the simple procedure of placing tissue in tubes, tightening the caps and placing tubes in the designated instrument position. Such a fast turnaround for sample processing would not be possible in a non-fluorophore and/or aqueous-phase procedure. The speed at which samples can be processed and the versatility in sample size and tissue type enables respiratory analysis that simply would not be feasible using other established approaches. The simultaneous measurement of many genotypes and the construction of multiple whole-plant respiratory budgets emphasise the potential of this method and its wider application.

## Additional file

**Additional file 1: Table S1.** Technical reproducibility of the fluorophore instrument in measuring the chemical oxidation of cysteine. **Figure S1.** The measured and expected depletion of O_2 _though replacement of air with known volumes of pure CO_2_ gas. **Figure S2.** A representative 46-day-old wheat plant harvested for whole-plant respiration analysis. **Figure S3.** Derived respiration rates in the dark (R_dark_) calculated from measurements between 5 and 20 min from experiment initiation using the various techniques.
