# Supplementary material for: The combination of gas-phase fluorophore technology and automation to enable high-throughput analysis of plant respiration
Source: Plant Methods. 2017 Mar 21;13:16. doi: 10.1186/s13007-017-0169-3 (PMC5361846; doi:10.1186/s13007-017-0169-3)
Supplement: Supplementary file 1 — Additional file 1: Table S1. Technical reproducibility of the fluorophore instrument in measuring the chemical oxidation of cysteine. Figure S1. The measured and expected depletion of O2 though replacement of air with known volumes of pure CO2 gas. Figure S2. A representative 46-day-old wheat plant harvested for whole-plant respiration analysis. Figure S3. Derived respiration rates in the dark (Rdark) calculated from measurements between 5 and 20 min from experiment initiation using the various techniques. [file 13007_2017_169_MOESM1_ESM.pdf]

Supporting Information

Table S1. Technical reproducibility of fluorophore instrument in measuring the chemical oxidation of cysteine across three separate experiments and for 30 sample tubes during each experiment.

	Experiment 1	Experiment 2	Experiment 3
O_2 consumption (% air h^{-1})	1.87	2.00	1.65
Standard deviation (% air h^{-1})	0.10	0.16	0.18
Coefficient of variation (%)	5.55	8.22	10.67

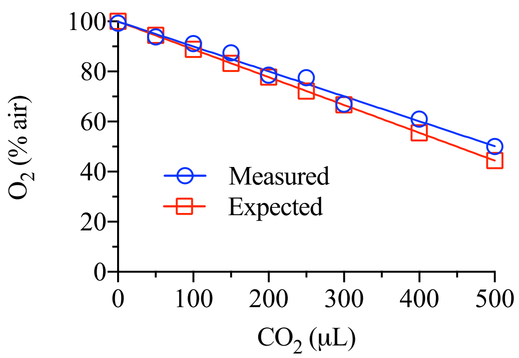

Figure S1. The measured (blue open circles) and expected (red open squares) depletion of O_2 (given as a % of O_2 relative to air) though replacement of air with known volumes of pure CO_2 gas in a 900 μL sample tube. The measured depletion values were linear (r^2=0.99) and had a slope of -0.0996 similar to the expected depletion rate of -0.1111.

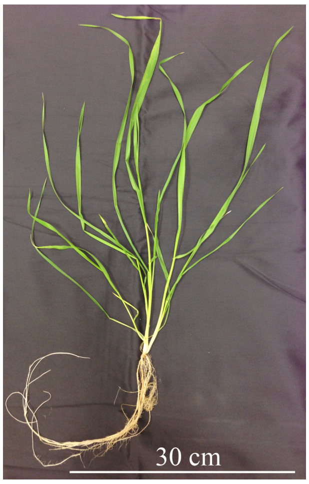

Figure S2. A representative 46-day-old wheat plant harvested for whole-plant respiration analysis by dissection into individual leaves, stem and roots and placement in sample tubes and analysis of O_2 consumption on the Q2 oxygen sensor.

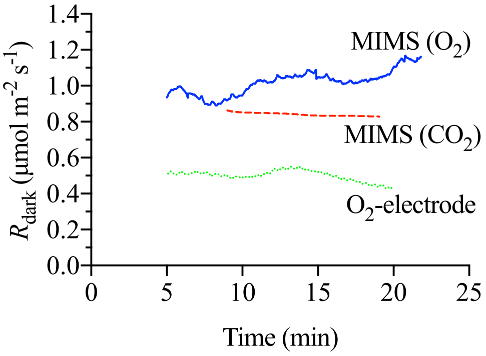

Figure S3. Derived respiration rates in the dark (R_{dark}) calculated from a 5 min running slope measured between 5 and 20 min from experiment initiation measured by membrane inlet mass spectrometry (MIMS) on an O_2 consumption (blue solid line), CO_2 evolution (red dashed line), or by O2-electrodes (dotted green line) after placing wheat leaf disks in the measuring cuvette.
